# Anatomy of the sacroiliac joint with relation to the lumbosacral trunk: Is there sufficient space for a two-hole plate?

**DOI:** 10.1371/journal.pone.0292620

**Published:** 2023-10-19

**Authors:** Michał Kułakowski, Karol Elster, Paweł Reichert, Aleksandra Królikowska, Joanna Jerominko, Paweł Ślęczka, Magdalena Grzonkowska, Michał Szpinda, Mariusz Baumgart

**Affiliations:** 1 Orthopaedic and Trauma Surgery Department, Independent Public Healthcare Center Rypin, Rypin, Poland; 2 Clinical Department of Trauma and Hand Surgery, Jan Mikulicz-Radecki University Clinical Hospital in Wroclaw, Wroclaw, Poland; 3 Ergonomics and Biomedical Monitoring Laboratory, Department of Physiotherapy, Faculty of Health Sciences, Wroclaw Medical University, Wroclaw, Poland; 4 Department of Radiology, Franciszek Lukaszczyk Oncology Center, Bydgoszcz, Poland; 5 Orthopaedic and Trauma Surgery Department, Independent Public Healthcare Center Myślenice, Myślenice, Poland; 6 Department of Normal Anatomy, The Ludwik Rydygier Collegium Medicum in Bydgoszcz, The Nicolaus Copernicus University in Toruń, Toruń, Poland; AIIMS: All India Institute of Medical Sciences, INDIA

## Abstract

Dislocations of the sacroiliac joint (SIJ) are treated with iliosacral screws or anterior plating. The study aimed to investigate the course of the lumbosacral trunk with reference to SIJ and determine whether is there sufficient space for two screws through the sacrum while performing anterior plating. Sixty patients, who underwent an MRI of the lumbar spine were included in our study. The three transverse LT—SIJ distances were measured at the three points (A, B, and C). We also analyzed 60 CT pelvic scans at points A, B, and C in order to measure: the vertebral canal–to–SIJ distance, the sacrum’s pelvic–to–dorsal surface sagittal distance, and the median plane–to–SIJ angle. The mean transverse LT–SIJ distances at points A, B, and C were 20.0 ± 3.05 mm, 17.9 ± 3.20 mm, and 12.3 ± 2.49 mm, respectively. Based on CT analyses, the vertebral canal–to–SIJ distances were 30.5 ± 7.65 mm at point A, 21.4 ± 5.05 mm at point B and 15.7 ± 6.05 mm at point C. The sacrum’s pelvic–to–dorsal surface sagittal distances reached values: 35.1 ± 11.62 mm at point A, 52.5 ± 10.58 mm at point B, and 57.5 ± 7.79 mm at point C. The median plane–to–SIJ angles measured 31.4 ± 4.82 degrees at point A, 26.6 ± 3.77 degrees at point B and 21.3 ± 3.25 mm at point C. Proximally, the safe zone for applying an anterior plate of SIJ is 20.0 mm. Since both the safe zone and safe corridor taper distally, surgeons may securely use one screw of gradually increased length towards the distal direction of SIJ, with inclination of 30 degrees in relation to the median plane of the lesser pelvis.

## Introduction

Acetabular and pelvic fractures account for 1.5% of all bony fractures in adults and constitute 2–5% of all bony fractures that require hospital admission [1, 2]. Pelvic injuries may involve minor lacerations or major fractures with devastating and life-threatening consequences. The mortality rate is considerable, especially in patients with hemodynamic instability. The management of pelvic traumas has markedly transformed throughout the last three decades with a significant improvement in outcomes [3]. Patients with unstable fractures indispensably require surgery that contributes to a better prognosis with a moderately low rate of complications [4]. The posterior part of pelvic ring affords some 60% of total pelvic stability [5]. As a result, stable fixation of the posterior part of pelvic ring is of relevance in function restoration.

There are different methods of fixation of SIJ presented in the professional literature: anterior plating, posterior plating, iliosacral screws and sacral bar fixation. If the anterior part of pelvic ring is supposed to be treated operatively together with SIJ, anterior plating of SIJ and simultaneous approaching both anterior and posterior parts of pelvic ring for easier reduction are preferred [6]. The anterior approach to SIJ was first described by Avila, so as to treat SIJ infections [7]. Despite a risk of iatrogenic injury to anterior branches of lumbar nerves L4 and L5, in cases when low back soft tissues are compromised, the anterior approach to SIJ is a good choice [8]. The anterior sacroiliac anatomy was described by Kellam and Simpson [9], who found anterior branch L5 to pass approximately 2–3 cm medially to SIJ. There are few studies to do with reciprocal relations between LT and SIJ. However, it is noteworthy that all the aforementioned autopsy studies referred to the course of anterior branches L4 and L5 in a relatively small group [10–12]. In anterior plating of SIJ, a trajectory of screw placement is also very important. When the inclination angle is relatively smaller, a screw easily enters SIJ, resulting in loss of stability. On the other hand, when the angle is relatively greater, a screw falls into either the vertebral canal or sacral foramen. Bai et al. [13] studied the course of anterior branches L4 and L5 in relation to SIJ and also focused on CT images to illustrate a surgical safe zone of anterior plating of SIJ.

Since previous studies have entirely been performed in autopsied specimens, in the present study we decided to analyze the course of the lumbosacral trunk (LT) in MRI images of patients in the supine position, which is consistent with that while performing an operative procedure. As presented by Gill et al. [14], a treatment method of open book injuries remains controversial, and subsequently we also attempted to answer the question if there is sufficient space for a two-hole plate in this area.

The objectives of our study were to evaluate the course of LT in relation to SIJ and to answer the question if there is sufficient space for two screws through the sacrum in anterior plating of SIJ. We also aimed to assess the optimum length and optimum angle of sacral screws in 2D CT scans.

## Material and methods

For the present study Ethics Committee Agreement of Kuyavian-Pomeranian Medical Chamber (13/KB/2021) was achieved.

All patients received and signed an informed consent document. Furthermore, no minors have been included in our study.

The sample size was calculated on the basis of a pilot study, assuming an estimator accuracy of 1 mm.

### 1. MRI measurements

We performed a prospective analysis of 60 consecutive patients, who underwent MRI of the lumbar spine in Lipno (Poland) for different pathological reasons.

**Fig 1:**
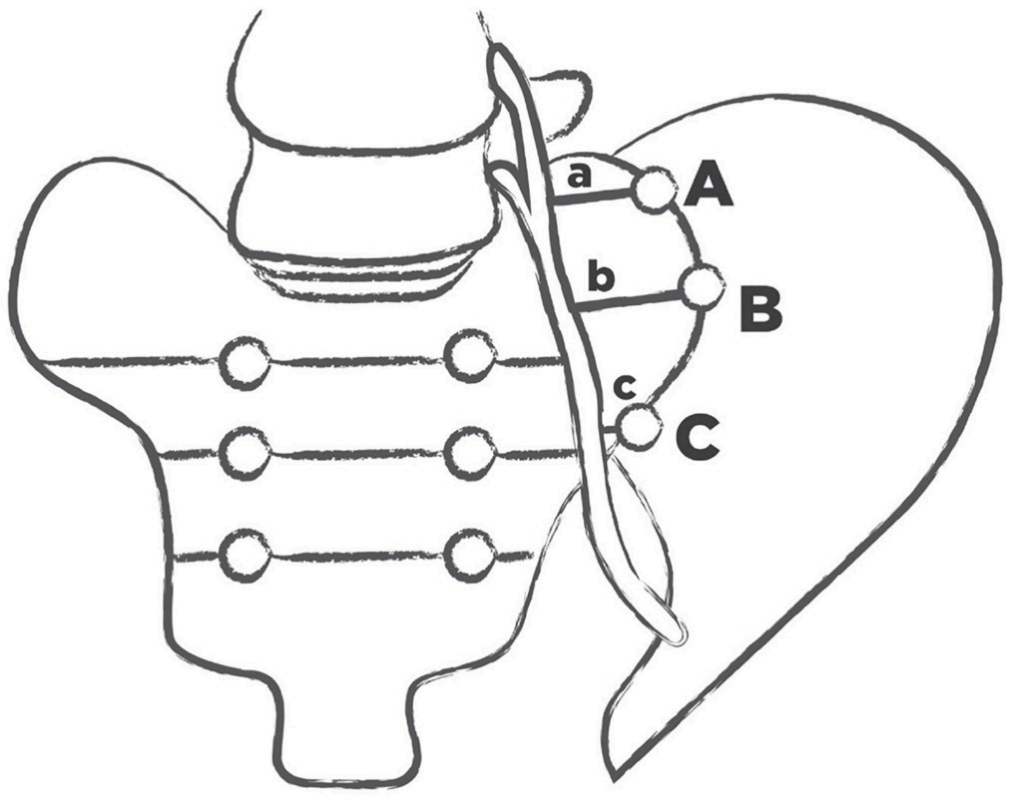
A diagram showing the anterior contour of the left SIJ and anterior branches L4 and L5 comprising LT. Point A: the highest point of SIJ, point C—the lowest point of SIJ and point B—the midpoint along SIJ. Stretches a, b and c correspond to the transverse LT—SIJ distances between respective points A, B or C and LT.

**Fig 2:**
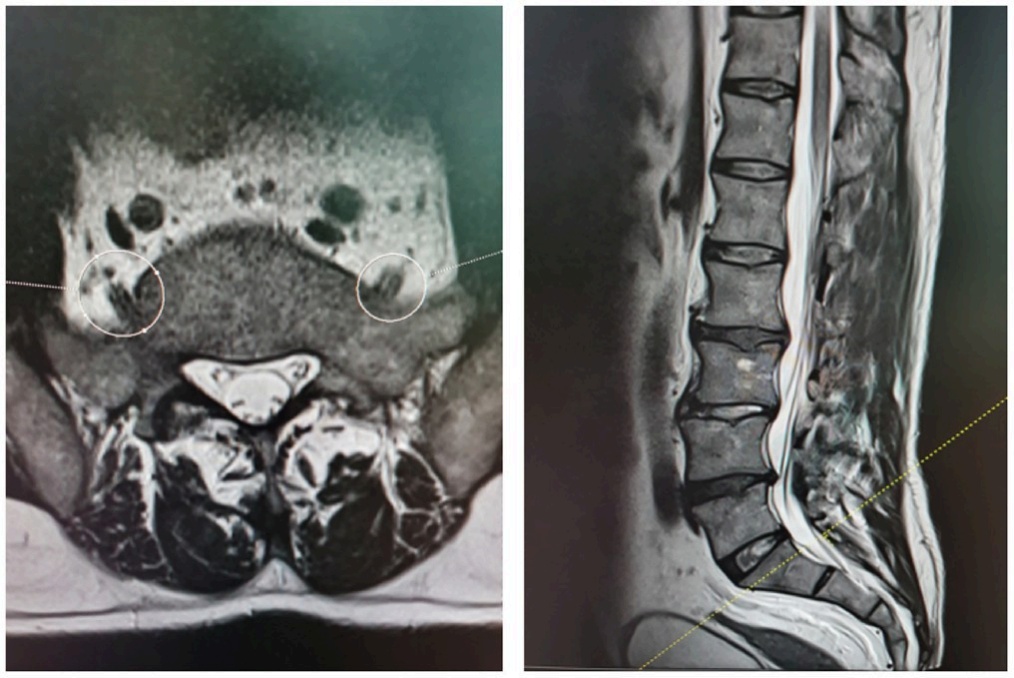
MRI showing transverse and sagittal projections with level of point A of SIJ.

**Fig 3:**
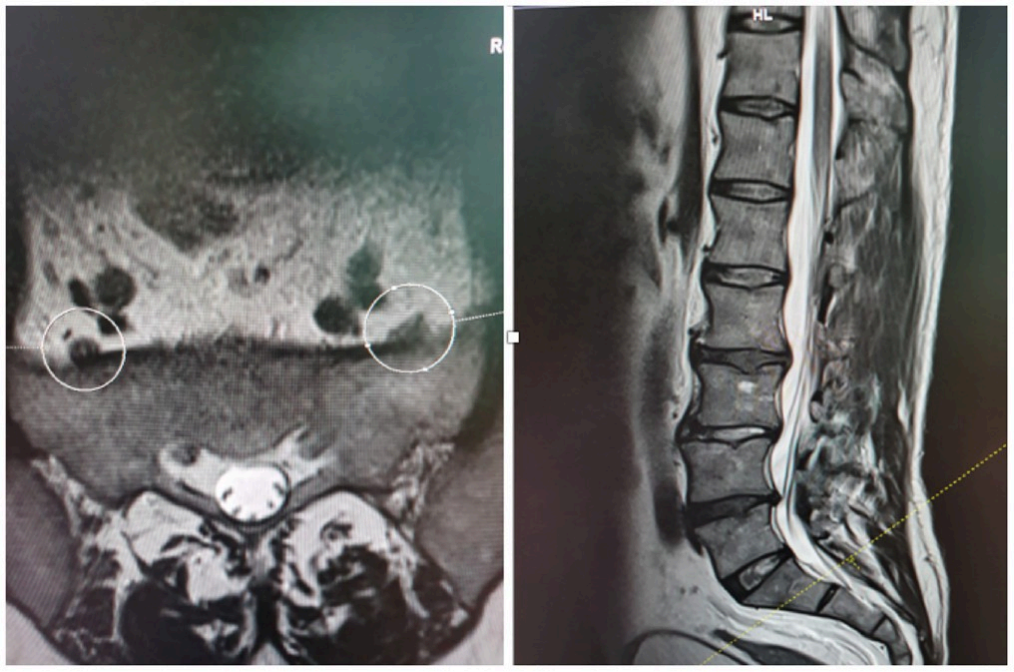
MRI showing transverse and sagittal projections with level of point B of SIJ.

**Fig 4:**
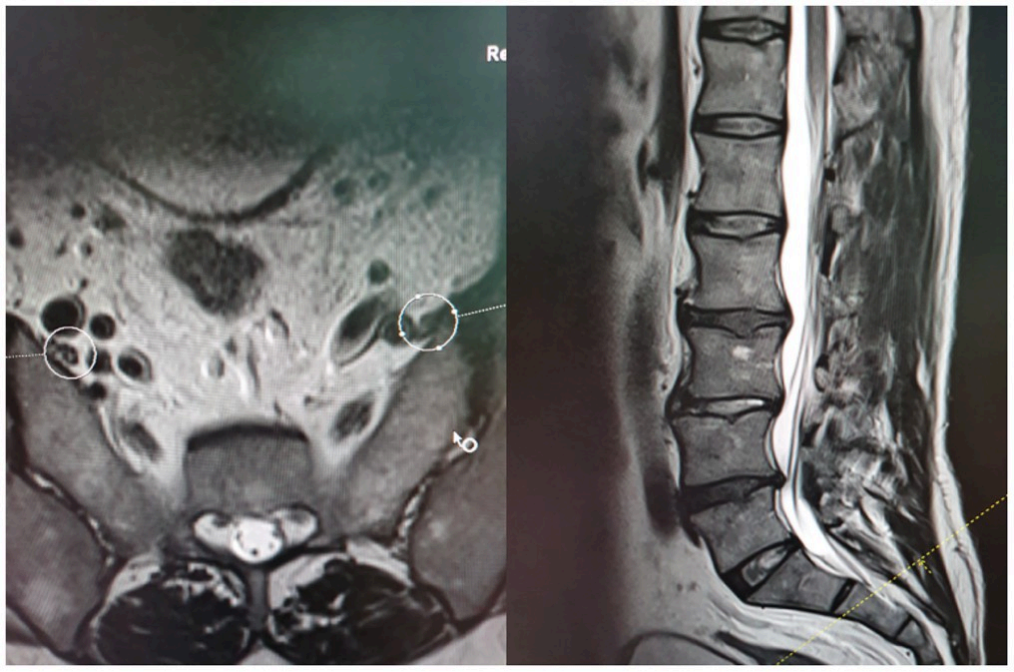
MRI showing transverse and sagittal projections with level of point C of SIJ.

The transverse distances (a, b and c) between anterior branches L4 and L5 and SIJ—called transverse LT—SIJ distances—at the three points A, B and C were measured. Points A, C and B referred to the highest point of SIJ, the lowest point of SIJ and the midway between points A and C, respectively (Figs 1–4).

### 2. CT measurements

We also prospectively analyzed CT scans of the pelvis that were performed because of different pathological reasons in 60 consecutive patients at the Independent Public Healthcare Hospital in Rypin.

**Fig 5:**
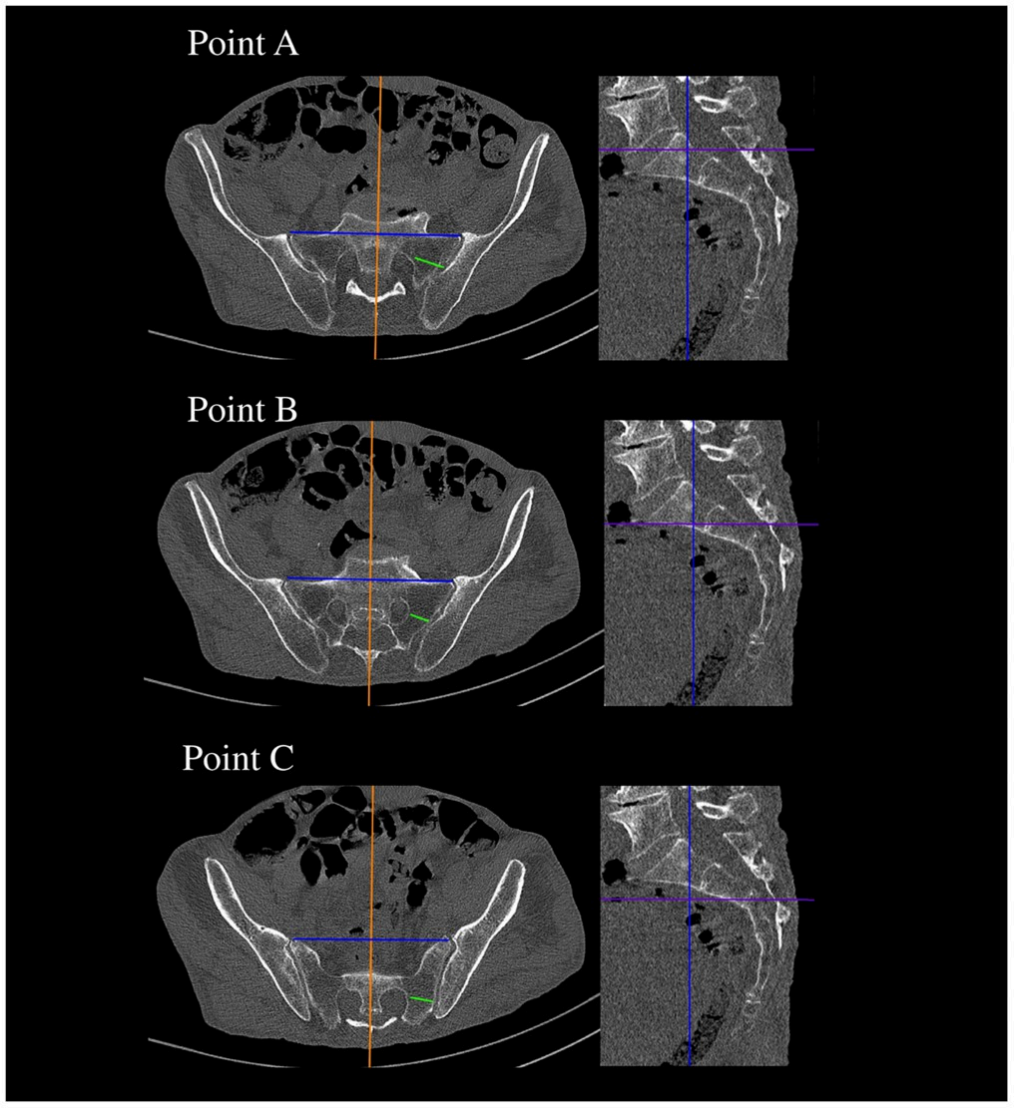
CT showing the widths between SIJ and the sacral canal that correspond to safe corridors for screws at points A, B, and C.

**Fig 6:**
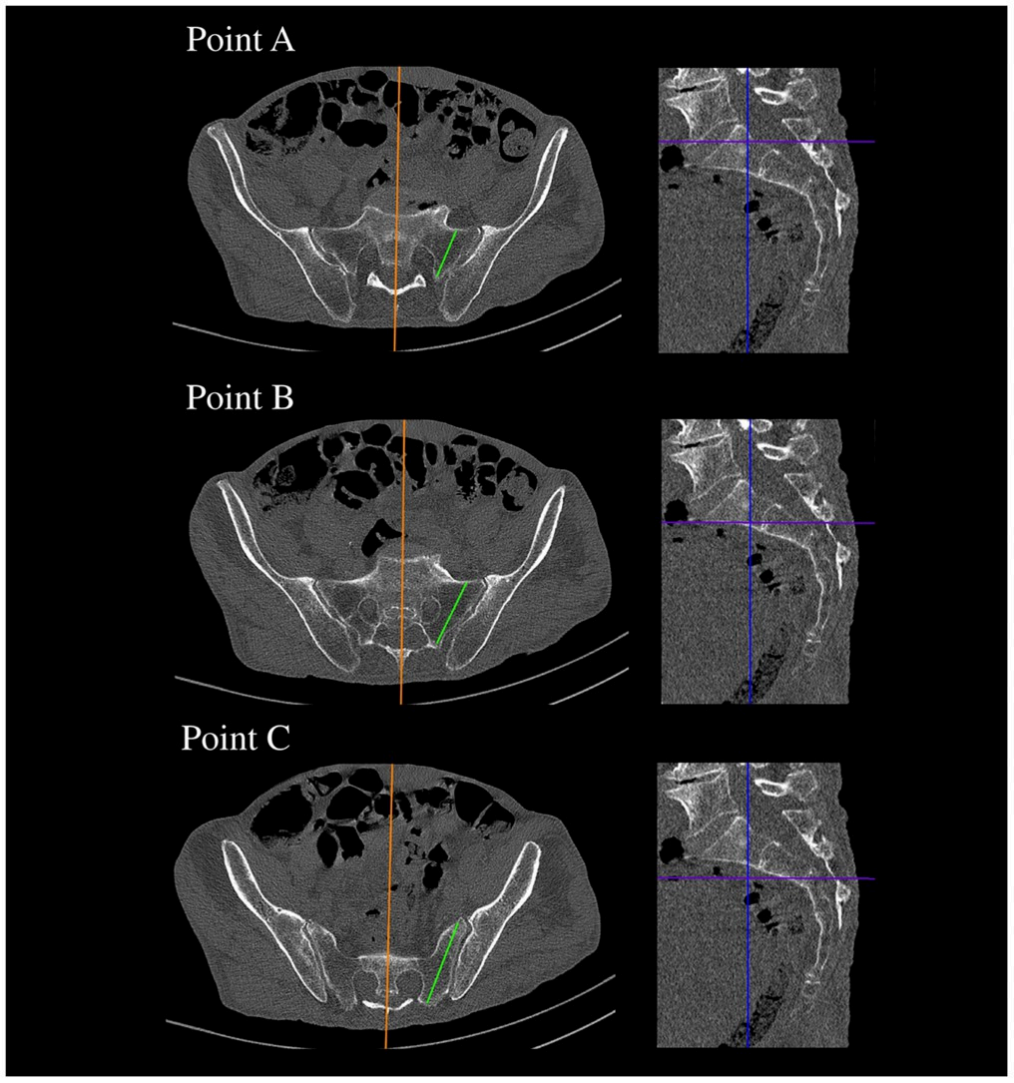
CT showing the sagittal distance between the pelvic and dorsal surfaces of sacrum that corresponds to the length of a screw, which can be applied at each level.

**Fig 7:**
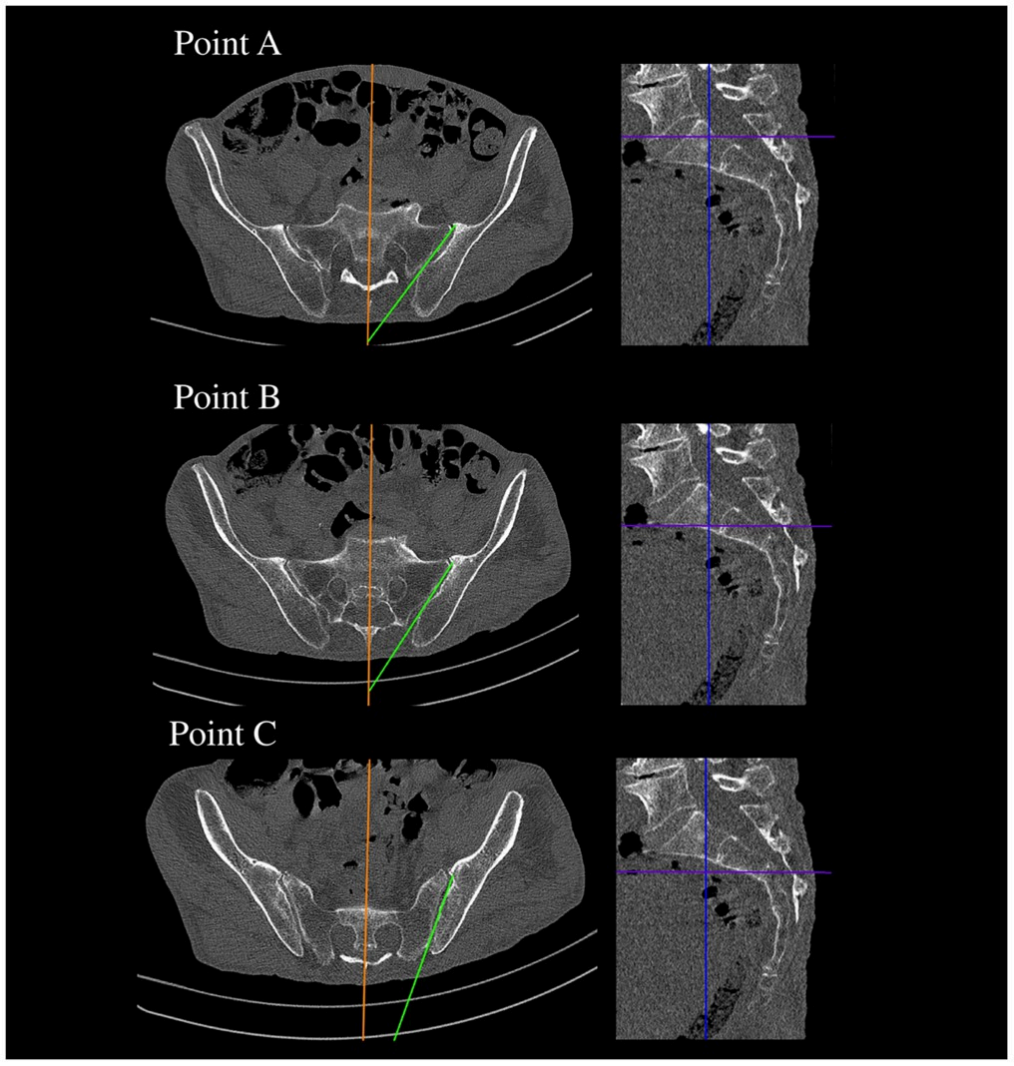
CT showing the angle between the median plane and SIJ—This measurement corresponds to the angle, at which a screw should be directed.

In the transverse projections with levels of the three points: A, B and C the following three parameters were measured:

1. the distance between the lateral edge of vertebral (sacral) canal or intervertebral foramen and the medial border of SIJ that was called the vertebral canal—to—SIJ distance (Fig 5); this measurement corresponds to the width of a safe corridor for screws at each level,
2. the sagittal distance between the pelvic and dorsal surfaces of sacrum, called the sacrum’s pelvic—to—dorsal surface sagittal distance (Fig 6); this measurement corresponds to the length of a screw, which can be used at each level, and
3. the angle between the median plane and SIJ, called the median plane—to—SIJ angle (Fig 7); this measurement corresponds to the angle, at which a screw should be directed.

### 3. Statistical analysis

In an effort to examine sex differences, Mann-Whitney U-test was used. Continuous variables have been presented as means and standard deviations (SD), while categorical data have been expressed as frequencies of appearance and percentages.

## Results

The findings have been presented as MRI measurements, CT measurements and dimensions of a pelvic plate.

Due to General Data Protection Regulation valid in every case when dealing with European Community Citizens personal data, we must have put a statement that our data would be available under request.

### 1. MRI measurements

MRI measurements referred to 44 women and 16 men of mean age 49.5 ± 13.3 years, with no significant age difference between two sexes.

The transverse LT—SIJ distances at points A, B and C have been presented in Table 1.

**Table 1:** The transverse LT—SIJ distances at points A, B and C.

Points	Mean transverse LT—SIJ distance ± SD (mm)	Mean transverse LT—SIJ distance ± SD in women (mm)	Mean transverse LT—SIJ distance ± SD in men (mm)	Sex differences P
A	20.0 ± 3.05	19.0 ± 3.73	21.0 ± 2.59	0.2
B	17.9 ± 3.20	18.2 ± 3.02	17.2 ± 3.60	0.1
C	12.3 ± 2.49	12.7 ± 1.96	12.0 ± 3.60	0.1

With no sex differences, the mean transverse LT—SIJ distances reached the following values: 20.0 ± 3.05 mm at point A, 17.9 ± 3.20 mm at point B and 12.3 ± 2.49 mm at point C.

### 2. CT measurements

CT measurements referred to 33 men and 27 women of mean age 58.1 ± 10.9 years, with no significant age difference between two sexes, presented in Table 2.

**Table 2:** The vertebral canal—To—SIJ distance (equal to the width of safe corridors) at points A, B and C.

Points	Mean vertebral canal—to—SIJ distance (mm)	Mean vertebral canal—to—SIJ distance in women (mm)	Mean vertebral canal—to—SIJ distance in men (mm)	Sex differences P
A	30.5 ± 7.65	28.7 ± 6.38	31.2 ± 7.68	0.19
B	21.4 ± 5.05	23.0 ± 7.06	19.8 ± 3.44	0.01
C	15.7 ± 6.05	16.9 ± 3.41	14.6 ±2.97	0.012

As presented in Table 2, the vertebral canal—to—SIJ distances at points B and C proved to be significantly greater in women than in men.

As presented in Table 3, the sacrum’s pelvic—to—dorsal surface sagittal distance at point C was significantly greater in men than in women.

**Table 3:** The sacrum’s pelvic—To—dorsal surface sagittal distance (equal to the length of a screw, which can be applied) at points A, B and C.

Points	Mean distance (mm)	Mean distance in women (mm)	Mean distance in men (mm)	Sex differences P
A	35.1 ± 11.62	34.8 ± 10.62	35.4 ± 12.05	0.8
B	52.5 ± 10.58	52.6 ± 6.76	52.4 ± 11.65	0.9
C	57.5 ± 7.79	55.0 ± 6.54	60.0 ± 7.88	0.008

As presented in Table 4, the median plane—to—SIJ angle at point A was significantly greater in men than in women.

**Table 4:** The median plane—To—SIJ angle (equal to the angle, at which a screw should be directed) at points A, B and C.

Points	Mean median plane—to—SIJ angle (in degrees)	Mean median plane—to—SIJ angle in women (in degrees)	Mean median plane—to—SIJ angle in men (in degrees)	Sex differences P
A	31.4 ± 4.82	29.5 ± 4.42	33.3 ± 4.59	0.002
B	26.6 ± 3.77	25.7 ± 3.35	27.5 ± 3.92	0.25
C	21.3 ± 3.25	21.1 ± 4.05	21.5 ± 4.42	0.7

### 3. Dimensions of a pelvic plate

**Fig 8:**
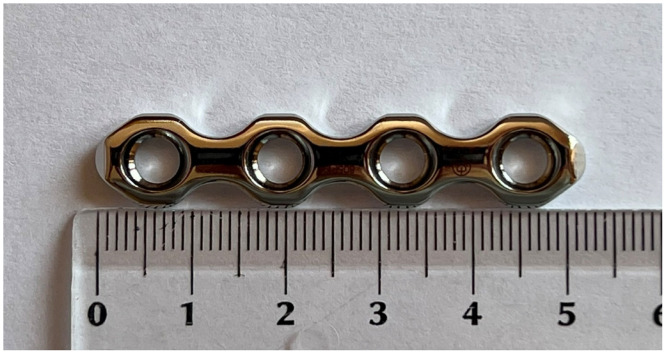
Dimensions of a pelvic plate.

**Fig 9:**
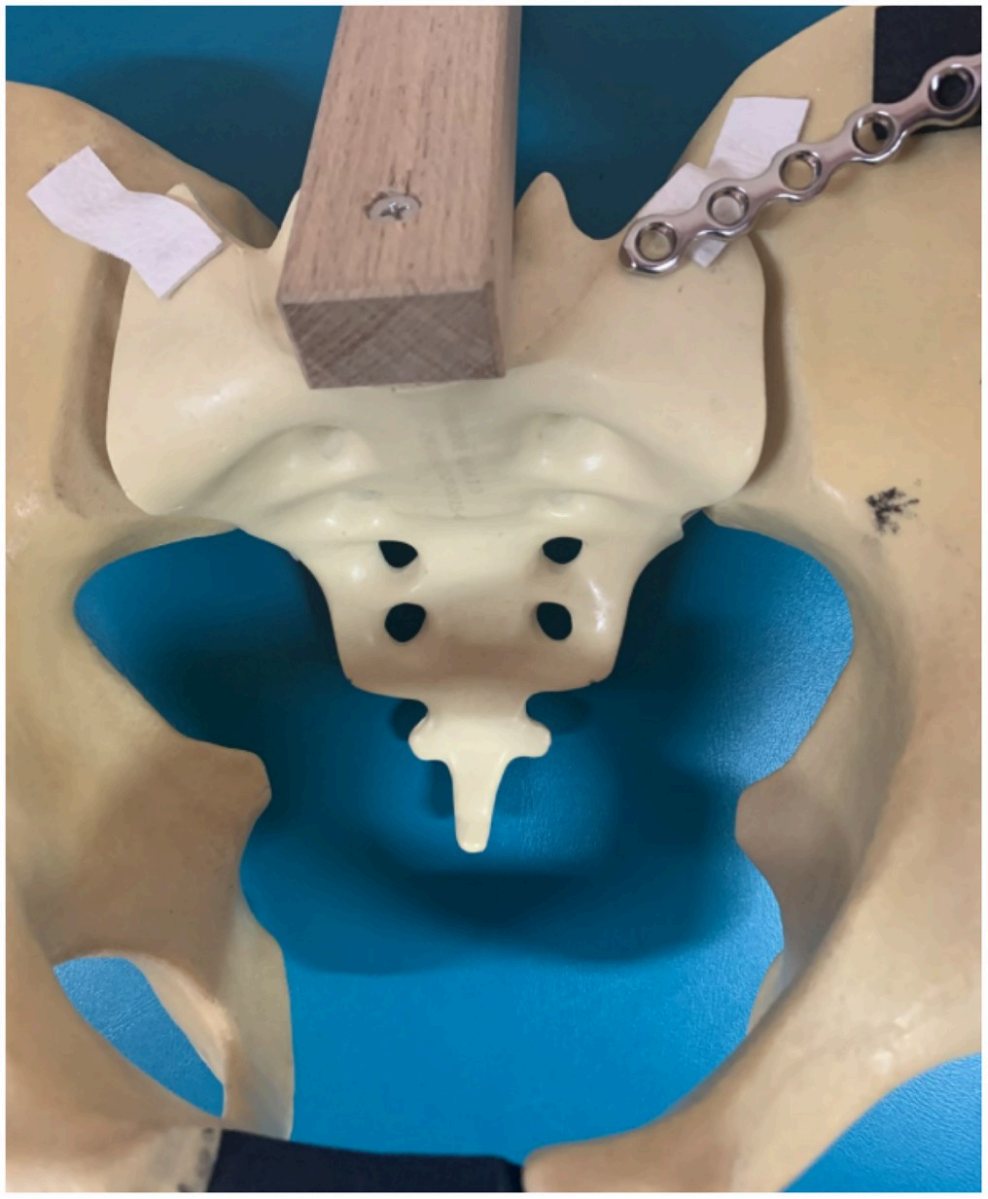
A pelvic plate applied on the anterior part of the sacrum.

In order to ensure if one can safely apply a two-hole plate on the sacrum here have been given dimensions of a pelvic plate (De Puy Synthes). The width measured from the border of a plate to the middle of the second hole is 20.0 mm (Figs 8 and 9).

## Discussion

In the present study the mean transverse LT—SIJ distances were as follows: 20.0 mm at point A, 17.9 mm at point B and 12.3 mm at point C. Our results remain consistent with the findings by Grechenig et al. [12], who examined the transverse LT—SIJ distances with level of the pelvic brim and in 1 cm intervals in length moving superiorly. From superior to inferior, their main three levels referred to the point of 86.3% of total length of SIJ, the point of 52.1% of total length of SIJ and the pelvic brim that corresponded to our points A, B and C, respectively. With relation to our points A—C, these authors found the transverse LT—SIJ distances to reach the following mean values: 21.25 mm at point A, 17.5 mm for ventral branch L4 and 19.8 mm for ventral branch L5 at point B, and 7.2 mm for ventral branch L4 and 9.1mm for ventral branch L5 at point C. Obviously, from a clinical point of view the most relevant is the smallest transverse LT—SIJ distance, which refers to point C and is even more reduced than that in our study.

With the use of 10 embalmed and 6 fresh autopsied specimens, Ebraheim et al. [10] measured the mean distances between anterior branches L4 or L5 and SIJ just 4 cm proximal to the linea terminalis. They found these distances to reach values 23 mm and 26 mm, respectively. It should be noticed that the aforementioned point projected between our points A and B, and besides the relevant distance between anterior branch L4 and SIJ is in line with our findings. Along the linea terminalis, which separates the false pelvis from the true pelvis the mean distance of 10 mm for both anterior branches L4 and L5 turned out to be consistent with our results.

Atlihan et al. [11] dissected 60 autopsied specimens to focus on the anterior branches of spinal nerves at risk during approach to the anterior aspect of SIJ. The mean distance between SIJ and the lateral border of anterior branch L5 on exiting the intervertebral foramen was 18.4 mm. Such a point projected somewhat superior to our point A. At the linea terminalis, the transverse LT—SIJ distance was on average 5.3 mm that was much less than in our study (12.3 mm).

Bai et al. [13] evaluated the transverse LT—SIJ distance in 15 autopsied adults preserved in 10% formalin. These authors revealed the transverse LT—SIJ distances to be 2.1 cm for ventral branch L4 and 2.6 cm for ventral branch L5 at point A, 1.7 cm for ventral branch L4 and 2.2 cm for ventral branch L5 at point B, and 1.2 cm for ventral branch L4 and 1.5 cm for ventral branch L5 at point C. Of note, these results strictly corresponded to our study. Bai et al. also assessed the vertical distance between anterior branches L4 or L5 and the pelvic surface of sacrum. These authors found anterior branch L5 to entirely pass on the pelvic surface of sacrum. The vertical distances between anterior branch L4 and points A, B or C were 9.5 cm, 5.6 cm and 0.2 cm respectively. Anterior branch L4 proved to be both longer and thinner than anterior branch L5. This may contribute to a much rare iatrogenic injury to anterior branch L4 in comparison to anterior branch L5. Also Leighton et al. [15] found a larger number of iatrogenic injuries to anterior branches L5. According to these authors, nerve fibers within anterior branches L5 were intensively packed by the dense fibrous connective tissue. Contrariwise, nerve fibers within anterior branches L4 indicated a much greater range of motion as the psoas major muscle separates the nerve from the pelvic surface of sacrum. These results are different from findings by Atlihan et al. [11], who found anterior branch L4 to be more susceptible to an intraoperative injury because of its closeness to SIJ.

It should be accentuated that all the aforementioned studies referred to measurements in cadavers. Most of these studies were based on embalmed specimens that could introduce potential bias of tissue distortion or tissue shrinkage. Contrariwise, our measurements in the present study were achieved in 60 patients, who underwent MRI. The study was performed in the supine position of patients that is similar to the patient’s position during surgery.

In the professional literature we found only two studies addressing sacral parameters in CT scans. Ebraheim et al. [10] found the anterior—posterior dimensions of SIJ with level of the pelvic brim and 1 cm medial to SIJ to be 6.0 cm and 4 cm above the pelvic brim to be only 5.0 cm. They showed a decrease in the bony stock in the upper part of SIJ that is in line with our findings. Since the anterior—posterior dimension of SIJ provides information how long a screw can be applied at each level, in our study lengths of screws at points A, B and C should reach the following values: 35.1 mm, 52.5 mm and 57.5 mm respectively. Ebraheim et al. [10] revealed the bony stock of the ilium to decrease rapidly just lateral to SIJ. At the point placed 2 cm lateral to SIJ such a distance was even less than 1 cm.

The study by Bai et al. [13] revealed the sacral bony stock to decrease in the posterior part of SIJ. They also assessed the angle between SIJ and the median plane that approximated 30°. Our study revealed the median plane—to—SIJ angle to decrease from 31.4° at point A to 21.3° at point C. We have concluded that screws in the distal part of SIJ should be placed more vertically. Besides, Bai et al. [13] assessed the vertical distances between SIJ and the spinal canal or the 1^st^ anterior sacral foramen that were 3.3 cm at point A and 2.0 cm at point C. We found that a safe corridor for screws was medially limited by the vertebral canal at point A and by the 1^st^ anterior sacral foramen at points B and C. The widths of safe corridors for screws were 30.5 mm at point A, 21.4 mm at point B and 15.7 mm at point C, respectively.

In CT measurements we observed considerable differences between patients that might be contributable to the pelvic size. As suggested by Krappinger et al. [16], a precise preoperative planning based on CT scans may minimize screw misplacement at SIJ.

The limitation of our MRI study was the evaluation of the course of anterior branch L5 alone. Our radiologist found the MRI evaluation of the course of anterior branch L4 very difficult and unreliable.

Another limitation of our study was that all of our participants were Caucasians and so further studies are needed to apply our findings according to ethnic differences.

The aim of our study was to answer the question if there is sufficient space for inserting two screws on the upper part of the sacrum during anterior plating of SIJ. Our results support findings of other authors that a surgeon can safely apply a plate in the upper sacrum with two screws, as displayed in Figs 8 and 9. Inserting two screws in the upper sacrum is important due to decreasing bone stock in this region.

## Conclusions

1. Proximally, the safe zone for applying an anterior plate of SIJ is 20.0 mm that is enough space for applying two screws in the sacrum with the widest safe corridor for inserting screws, and besides a surgeon may use the shortest screw at point A.
2. Since both the safe zone and safe corridor taper distally, surgeons may securely use one screw of gradually increased length towards the distal direction of SIJ.
3. With relation to the median plane of the lesser pelvis a screw in the sacrum is advised to incline medially at an angle of 30 degrees.

## Supporting information

S1 Appendix Data generated and analyzed during the present study. MRI measurement.
(PDF)

S2 Appendix CT measurements. (PDF)
