# Correction: Anatomy of the sacroiliac joint with relation to the lumbosacral trunk: Is there sufficient space for a two-hole plate?

**DOI:** 10.1371/journal.pone.0318384

**Published:** 2025-01-24

**Authors:** Michał Kułakowski, Karol Elster, Paweł Reichert, Aleksandra Królikowska, Joanna Jerominko, Paweł Ślęczka, Magdalena Grzonkowska, Michał Szpinda, Mariusz Baumgart

There is an error in affiliation 2 for author Paweł Reichert. The correct affiliation 2 is: Department of Orthopedics, Traumatology and Hand Surgery, Faculty of Medicine, Wroclaw Medical University, Wroclaw, Poland.
