# Supplementary material for: Anatomy of the sacroiliac joint with relation to the lumbosacral trunk: Is there sufficient space for a two-hole plate?
Source: PLoS One. 2023 Oct 19;18(10):e0292620. doi: 10.1371/journal.pone.0292620 (PMC10586703; doi:10.1371/journal.pone.0292620)
Supplement: S1 Appendix — MRI measurement. (PDF) [file pone.0292620.s001.pdf]

Suplement file 1
Data generated and analyzed during the present study.
MRI measurement

Number	sex	age	Point	Right side (mm)	Left side (mm)
1	F	22	A	23	26
			B	17	17
			C	10	10
2	F	47	A	16	22
			B	13	12
			C	11	6
3	F	79	A	18	18
			B	19	17
			C	13	13
4	F	47	A	24	27
			B	20	22
			C	15	13
5	M	34	A	26	27
			B	18	21
			C	12	13
6	F	54	A	23	22
			B	18	21
			C	10	12
7	M	56	A	20	21
			B	17	19
			C	11	13
8	M	63	A	19	18
			B	15	15
			C	11	7
9	F	36	A	22	23
			B	14	18
			C	10	13
10	F	48	A	26	25
			B	18	20
			C	13	15
11	M	43	A	20	20
			B	17	15
			C	13	10
12	F	42	A	20	23
			B	19	28
			C	15	16
13	F	66	A	17	20
			B	14	23

			C	12	22
14	F	37	A	24	24
			B	20	16
			C	11	12
15	M	71	A	21	21
			B	18	21
			C	13	13
16	F	50	A	26	26
			B	21	19
			C	13	10
17	M	53	A	27	29
			B	28	26
			C	24	21
18	F	43	A	24	24
			B	20	21
			C	14	8
19	F	41	A	27	26
			B	17	23
			C	14	15
20	F	56	A	20	26
			B	15	23
			C	11	17
21	M	39	A	21	23
			B	18	18
			C	11	13
22	F	58	A	24	23
			B	20	20
			C	10	8
23	F	40	A	27	28
			B	17	21
			C	13	10
24	M	49	A	21	17
			B	16	12
			C	10	9
25	F	81	A	22	19
			B	23	20
			C	13	16
26	M	34	A	25	27
			B	17	19
			C	8	11
27	M	50	A	18	20
			B	13	14
			C	9	10
28	M	51	A	20	24
			B	18	20
			C	10	14

29	F	61	A	21	23
			B	19	21
			C	16	16
30	F	17	A	23	20
			B	15	15
			C	11	12
31	F	50	A	23	21
			B	20	17
			C	13	15
32	F	45	A	24	23
			B	17	21
			C	14	13
33	F	50	A	25	25
			B	20	18
			C	13	10
34	F	56	A	18	19
			B	20	17
			C	15	13
35	F	69	A	20	20
			B	13	11
			C	10	7
36	F	46	A	18	15
			B	13	10
			C	11	10
37	F	40	A	25	21
			B	23	13
			C	15	11
38	F	68	A	23	19
			B	25	22
			C	15	12
39	M	44	A	26	28
			B	21	19
			C	12	13
40	F	19	A	30	26
			B	20	18
			C	14	11
41	M	47	A	21	15
			B	12	10
			C	11	10
42	F	21	A	23	24
			B	19	22
			C	13	12
43	M	53	A	21	18
			B	14	16
			C	10	10
44	F	59	A	21	20

			B	16	13
			C	12	10
45	F	66	A	24	21
			B	16	18
			C	13	9
46	F	53	A	22	21
			B	14	18
			C	10	13
47	F	52	A	23	20
			B	19	17
			C	13	11
48	F	45	A	23	24
			B	19	16
			C	13	11
49	F	32	A	21	17
			B	19	16
			C	15	14
50	F	50	A	27	25
			B	21	26
			C	11	16
51	F	43	A	15	19
			B	17	15
			C	11	10
52	M	71	A	21	15
			B	17	13
			C	11	9
53	M	67	A	23	22
			B	21	21
			C	14	13
54	F	54	A	21	20
			B	16	14
			C	11	12
55	F	50	A	18	22
			B	17	18
			C	12	19
56	F	34	A	23	18
			B	13	12
			C	10	7
57	F	69	A	19	20
			B	25	29
			C	19	19
58	F	45	A	21	20
			B	18	16
			C	12	12
59	F	50	A	23	23
			B	22	22

			C	14	12
60	M	53	A	18	20
			B	18	15
			C	15	12
