# Supplementary material for: Anatomy of the sacroiliac joint with relation to the lumbosacral trunk: Is there sufficient space for a two-hole plate?
Source: PLoS One. 2023 Oct 19;18(10):e0292620. doi: 10.1371/journal.pone.0292620 (PMC10586703; doi:10.1371/journal.pone.0292620)
Supplement: S2 Appendix — (PDF) [file pone.0292620.s002.pdf]

Supplement 2
CT measurements

Table 1.

The vertebral canal – to – SIJ distance (equal to the width of safe corridors) at points A. B and C. (in milimeters)

Patient's number	sex	**channel A-L**	**channel A-R**	**Channel B-L**	**channel B-R**	**channel C-L**	**channel C-R**
1	m	**19.8**	**30.1**	**30.4**	**31.8**	**25.8**	**27.2**
2	m	**31.7**	**32.6**	**31.8**	**32.6**	**26.2**	**28.6**
3	m	**39.1**	**33.9**	**31.5**	**35.1**	**11.5**	**15.6**
4	m	**38.7**	**36.8**	**17.4**	**16.8**	**14.9**	**10**
5	m	**35.3**	**32.4**	**27.9**	**29.4**	**26**	**26.9**
6	f	**34.8**	**39.5**	**25.2**	**27.2**	**25.6**	**25.3**
7	m	**30.7**	**36.6**	**33.2**	**32.6**	**28.5**	**26.9**
8	f	**36.2**	**36.6**	**32.2**	**27.9**	**28.2**	**28.8**
9	m	**38**	**42.2**	**28.7**	**29.3**	**29.2**	**28.9**
10	m	**42.2**	**40.7**	**27.2**	**35.1**	**20.5**	**19.8**
11	m	**37.6**	**33.7**	**26**	**20.6**	**19.2**	**12.7**
12	m	**46**	**42.8**	**26**	**27.3**	**17.1**	**22.2**
13	m	**43.4**	**42.6**	**19.8**	**23**	**25.2**	**26.5**
14	m	**22.1**	**25.6**	**22.6**	**20.7**	**29.6**	**29.2**
15	m	**39.2**	**39.2**	**26.2**	**24.5**	**23**	**28.4**
16	m	**34.2**	**30.6**	**20.8**	**19.9**	**31.7**	**30**
17	m	**18.7**	**22.3**	**22.8**	**20.1**	**29.7**	**31.6**
18	m	**34.1**	**33.8**	**21**	**19.4**	**34.1**	**32.6**
19	f	**30.4**	**29.7**	**17.6**	**8.1**	**20.7**	**21.8**
20	f	**29**	**32.4**	**26.1**	**29.2**	**24.6**	**28.8**
21	f	**28.1**	**33.9**	**22.8**	**24.3**	**30.2**	**32.5**
22	f	**24.4**	**25.3**	**24.3**	**24.8**	**21.1**	**22.6**
23	f	**34.1**	**29.6**	**21.5**	**19.1**	**33.1**	**1.8**
24	f	**20.7**	**20.8**	**22.4**	**22.4**	**30.7**	**29.7**
25	f	**39.4**	**39.6**	**26.8**	**24.5**	**25.1**	**19.1**
26	f	**21.9**	**28.5**	**26.8**	**26.8**	**29**	**30.9**
27	f	**25**	**17.3**	**21.4**	**20.7**	**32.9**	**30.5**
28	f	**21.5**	**21.9**	**27**	**17.9**	**32**	**30.3**
29	f	**25.4**	**17.6**	**25.3**	**19.1**	**31**	**21.1**
30	f	**26.1**	**26.1**	**20.4**	**20.3**	**28.7**	**26**
31	f	**23.4**	**26**	**25.4**	**25**	**31**	**31.8**
32	f	**27.1**	**22.5**	**30.4**	**25.6**	**19.2**	**21.1**
33	f	**26.4**	**29.1**	**21.5**	**22.4**	**28.9**	**25.5**
34	f	**16**	**16**	**20.5**	**21.3**	**17.6**	**18.1**
35	f	**28.6**	**23.6**	**18.5**	**15.9**	**31.8**	**30.3**
36	f	**27.6**	**28.1**	**19.7**	**16.8**	**30.1**	**29.7**

37	f	**21.5**	**21.9**	**27**	**17.9**	**32**	**30.3**
38	f	**25.4**	**17.6**	**25.3**	**19.1**	**31**	**21.1**
39	f	**26.1**	**26.1**	**20.4**	**20.3**	**28.7**	**26**
40	f	**37.6**	**33.7**	**26**	**20.6**	**19.2**	**12.7**
41	f	**46**	**42.8**	**26**	**27.3**	**17.1**	**22.2**
42	f	**43.4**	**42.6**	**19.8**	**23**	**25.2**	**26.5**
43	f	**35.3**	**32.4**	**27.9**	**29.4**	**26**	**26.9**
44	f	**34.8**	**39.5**	**25.2**	**27.2**	**26.6**	**26.7**
45	f	**22.1**	**25.6**	**22.6**	**20.7**	**29.6**	**29.2**
46	f	**29**	**32.4**	**26.1**	**29.2**	**24.6**	**28.8**
47	f	**27.1**	**22.5**	**30.4**	**25.6**	**19.2**	**21.1**
48	f	**46**	**42.8**	**26**	**27.3**	**17.1**	**22.2**
49	f	**27.1**	**22.5**	**30.4**	**25.6**	**19.2**	**21.1**
50	f	**31.7**	**32.6**	**31.8**	**32.6**	**26.2**	**28.6**
51	f	**30.7**	**36.6**	**33.2**	**32.6**	**28.5**	**26.9**
52	f	**31.7**	**32.6**	**31.8**	**32.6**	**26.2**	**28.6**
53	f	**34.8**	**39.5**	**25.2**	**27.2**	**29.2**	**28.5**
54	f	**34.8**	**39.5**	**25.2**	**27.2**	**27.8**	**28.8**
55	f	**35.3**	**32.4**	**27.9**	**29.4**	**26**	**26.9**
56	f	**22.1**	**25.6**	**22.6**	**20.7**	**29.6**	**29.2**
57	f	**26.4**	**29.1**	**21.5**	**22.4**	**28.9**	**25.5**
58	f	**46**	**42.8**	**26**	**27.3**	**17.1**	**22.2**
59	f	**42.2**	**40.7**	**27.2**	**35.1**	**20.5**	**19.8**
60	f	**38**	**42.2**	**28.7**	**29.3**	**29.2**	**28.9**

Table 2. The sacrum's pelvic – to – dorsal surface sagittal distance (equal to the length of a screw. which can be applied) at points A. B and C.

Patient's number	sex	**Lenght A-L**	**Lenght A-R**	**lenght B-L**	**lenhgt B-R**	**lenght C-L**	**lenght C-R**
1	m	**18.5**	**19.3**	**50.2**	**44.3**	**59.8**	**58.1**
2	m	**18.2**	**19.1**	**50.4**	**42.1**	**61.2**	**57.4**
3	m	**13.6**	**17.5**	**18.5**	**22.9**	**26.8**	**39.8**
4	m	**13.2**	**18.6**	**42.7**	**36.1**	**58.2**	**59.8**
5	m	**17.5**	**31.5**	**48.8**	**48.6**	**61.5**	**66.4**
6	f	**11.8**	**20.2**	**35.3**	**40.1**	**40.5**	**49.6**
7	m	**18.1**	**19.2**	**51.4**	**41.7**	**61.2**	**57.3**
8	f	**12.3**	**17.6**	**51.4**	**52.3**	**52.8**	**53.7**
9	m	**10.8**	**12.7**	**42.5**	**37.1**	**56.3**	**64.2**
10	m	**42.2**	**40.7**	**35.1**	**27.2**	**52.3**	**58.6**
11	m	**24.2**	**39.7**	**56.8**	**59.2**	**66.7**	**67.4**
12	m	**40.7**	**37.3**	**60.9**	**61.6**	**68.9**	**67.6**
13	m	**53.4**	**48.3**	**66.3**	**58.4**	**55.9**	**51.9**
14	m	**42.3**	**37.4**	**53.7**	**51.1**	**61.5**	**63.7**
15	m	**43.8**	**52**	**55.8**	**62.5**	**72.6**	**66.6**

16	m	**44.5**	**47.7**	**66.5**	**67.5**	**66.6**	**66.6**
17	m	**48.3**	**42.7**	**63.6**	**62.1**	**61.2**	**66.3**
18	m	**48.5**	**45.6**	**45.2**	**48.4**	**55.2**	**58.9**
19	f	**35.4**	**39.4**	**55.6**	**55.5**	**65.3**	**65.6**
20	f	**39.4**	**40.8**	**49.8**	**46.3**	**59.7**	**55.7**
21	f	**46.3**	**41.8**	**71.1**	**71.5**	**70.4**	**75.4**
22	f	**45.4**	**41.5**	**50.4**	**53**	**51.2**	**51.4**
23	f	**42.8**	**38.1**	**40.4**	**50.3**	**56.4**	**58.9**
24	f	**42.2**	**40.6**	**62.2**	**62.6**	**62.3**	**62.7**
25	f	**42.2**	**45.7**	**57.5**	**60.2**	**61.9**	**62.3**
26	f	**40.1**	**41.1**	**55.1**	**56.3**	**61.9**	**62.8**
27	f	**44**	**44.6**	**59.8**	**61.6**	**51.7**	**56.3**
28	f	**40.2**	**36.9**	**67.2**	**66.2**	**64.7**	**57.2**
29	f	**40.8**	**40.6**	**59.1**	**54.5**	**54.7**	**64.7**
30	f	**42**	**39**	**54.3**	**54.4**	**58.5**	**59.7**
31	f	**35.6**	**44.9**	**56.8**	**62.6**	**63.3**	**53.9**
32	f	**36**	**35.5**	**43.7**	**49.2**	**50.7**	**51.9**
33	f	**41.1**	**33.7**	**48.9**	**51.8**	**48.7**	**57.6**
34	f	**45.5**	**46.7**	**55.3**	**58.4**	**65.6**	**66.1**
35	f	**35.1**	**35.8**	**56.8**	**54.6**	**50.9**	**52.5**
36	f	**34.8**	**33.9**	**54.4**	**54.6**	**50.2**	**49.9**
37	f	**40.2**	**36.9**	**67.2**	**66.2**	**64.7**	**57.2**
38	f	**40.8**	**40.6**	**59.1**	**54.5**	**54.7**	**64.7**
39	f	**42**	**39**	**54.3**	**54.4**	**58.5**	**59.7**
40	f	**24.2**	**39.7**	**56.8**	**59.2**	**66.7**	**67.4**
41	f	**40.7**	**37.3**	**60.9**	**61.6**	**68.9**	**67.6**
42	f	**53.4**	**48.3**	**66.3**	**58.4**	**55.9**	**51.9**
43	f	**17.5**	**31.5**	**48.8**	**48.6**	**61.5**	**66.4**
44	f	**11.8**	**20.2**	**35.3**	**40.1**	**40.5**	**49.6**
45	f	**42.3**	**37.4**	**53.7**	**51.1**	**61.5**	**63.7**
46	f	**39.4**	**40.8**	**49.8**	**46.3**	**59.7**	**55.7**
47	f	**36**	**35.5**	**43.7**	**49.2**	**50.7**	**51.9**
48	f	**40.7**	**37.3**	**60.9**	**61.6**	**68.9**	**67.6**
49	f	**36**	**35.5**	**43.7**	**49.2**	**50.7**	**51.9**
50	f	**18.2**	**19.1**	**50.4**	**42.1**	**61.2**	**57.4**
51	f	**18.1**	**19.2**	**51.4**	**41.7**	**61.2**	**57.3**
52	f	**18.2**	**19.1**	**50.4**	**42.1**	**61.2**	**57.4**
53	f	**11.8**	**20.2**	**35.3**	**40.1**	**40.5**	**49.6**
54	f	**11.8**	**20.2**	**35.3**	**40.1**	**40.5**	**49.6**
55	f	**17.5**	**31.5**	**48.8**	**48.6**	**61.5**	**66.4**
56	f	**42.3**	**37.4**	**53.7**	**51.1**	**61.5**	**63.7**
57	f	**41.1**	**33.7**	**48.9**	**51.8**	**48.7**	**57.6**
58	f	**40.7**	**37.3**	**60.9**	**61.6**	**68.9**	**67.6**
59	f	**42.2**	**40.7**	**35.1**	**27.2**	**52.3**	**58.6**
60	f	**10.8**	**12.7**	**42.5**	**37.1**	**56.3**	**64.2**

Table 3. The median plane – to – SIJ angle (equal to the angle. at which a screw should be directed) at points A. B and C (in milimeters).

Patient's number	sex	**angle A-L**	**angle A-R**	**angle B-L**	**angle B-R**	**angle C-L**	**angle C-R**
1	m	**30.12**	**34.4**	**22.9**	**25.1**	**10**	**11.5**
2	m	**30.6**	**33.5**	**26.2**	**26.7**	**12.9**	**18.4**
3	m	**33.4**	**32.8**	**21.6**	**33.4**	**19.1**	**15.4**
4	m	**31.4**	**33.1**	**24.2**	**24.4**	**22.2**	**22.9**
5	m	**30.3**	**29.9**	**26.5**	**19.6**	**21.1**	**18.8**
6	f	**30.4**	**28.7**	**27.8**	**26.8**	**24.4**	**21.3**
7	m	**36.9**	**37.4**	**31.6**	**28.7**	**21.5**	**21.7**
8	f	**26.4**	**23**	**22.2**	**21.9**	**22.2**	**20.2**
9	m	**30.8**	**29.5**	**19.9**	**19.4**	**20.8**	**19.5**
10	m	**29.8**	**25.5**	**23.7**	**20.2**	**21.7**	**16.2**
11	m	**32.2**	**33.8**	**26**	**25.5**	**22.1**	**21.9**
12	m	**35.2**	**34.4**	**29.4**	**27.6**	**24.3**	**21.9**
13	m	**39.7**	**39.5**	**29.6**	**33**	**24.9**	**25**
14	m	**30.2**	**29.9**	**28**	**26.5**	**19.3**	**20.8**
15	m	**26.9**	**26.6**	**24.6**	**24.4**	**20**	**19.1**
16	m	**39.4**	**40**	**22**	**26.8**	**27.5**	**27.9**
17	m	**35.3**	**32.8**	**31.8**	**27.5**	**23.9**	**21.7**
18	m	**27.2**	**28.9**	**22.9**	**22.6**	**18.9**	**19**
19	f	**40.2**	**36.7**	**32.0**	**30.8**	**24.1**	**24.4**

20	f	**39.5**	**41.7**	**30.2**	**29.2**	**27.7**	**27.7**
21	f	**37**	**30**	**29.4**	**26.8**	**20.9**	**20.6**
22	f	**30.2**	**26.6**	**25.6**	**26.3**	**19.3**	**16.9**
23	f	**42.5**	**41.1**	**34.1**	**36.5**	**31.6**	**28.7**
24	f	**24.2**	**24.1**	**23.2**	**23.2**	**17.2**	**17.1**
25	f	**30.3**	**30.9**	**29.5**	**30.2**	**20.1**	**20.6**
26	f	**24.1**	**28.6**	**26.1**	**26.2**	**18.4**	**19.8**
27	f	**31.5**	**30.4**	**25.9**	**27**	**18.7**	**20**
28	f	**34.2**	**36.8**	**25.6**	**28.3**	**24.5**	**24.1**
29	f	**36.1**	**36.3**	**23.2**	**23.1**	**23.3**	**23.4**
30	f	**30.4**	**31.5**	**28.9**	**28.8**	**21.7**	**22.9**
31	f	**29.4**	**31.4**	**29.6**	**29.6**	**20.1**	**20.9**
32	f	**27.8**	**27.8**	**20.1**	**20.3**	**17.9**	**16.7**
33	f	**30.3**	**32.1**	**31.1**	**31.3**	**21.4**	**20.8**
34	f	**26.7**	**23.7**	**24.4**	**20.4**	**17**	**16.3**
35	f	**38.5**	**37.6**	**28.5**	**31.2**	**31.3**	**27.3**
36	f	**36.3**	**36.8**	**27.9**	**28.2**	**32.8**	**29.9**
37	f	**34.2**	**36.8**	**25.6**	**28.3**	**24.5**	**24.1**
38	f	**36.1**	**36.3**	**23.2**	**23.1**	**23.3**	**23.4**
39	f	**30.4**	**31.5**	**28.9**	**28.8**	**21.7**	**22.9**
40	f	**32.2**	**33.8**	**26**	**25.5**	**22.1**	**21.9**
41	f	**35.2**	**34.4**	**29.4**	**27.6**	**24.3**	**21.9**
42	f	**39.7**	**39.5**	**29.6**	**33**	**24.9**	**25**

43	f	**30.3**	**29.9**	**26.5**	**19.6**	**21.1**	**18.8**
44	f	**30.4**	**28.7**	**27.8**	**26.8**	**24.4**	**21.3**
45	f	**30.2**	**29.9**	**28**	**26.5**	**19.3**	**20.8**
46	f	**39.5**	**41.7**	**30.2**	**29.2**	**27.7**	**27.7**
47	f	**27.8**	**27.8**	**20.1**	**20.3**	**17.9**	**16.7**
48	f	**35.2**	**34.4**	**29.4**	**27.6**	**24.3**	**21.9**
49	f	**27.8**	**27.8**	**20.1**	**20.3**	**17.9**	**16.7**
50	f	**30.6**	**33.5**	**26.2**	**26.7**	**12.9**	**18.4**
51	f	**36.9**	**37.4**	**31.6**	**28.7**	**21.5**	**21.7**
52	f	**30.6**	**33.5**	**26.2**	**26.7**	**12.9**	**18.4**
53	f	**30.4**	**28.7**	**27.8**	**26.8**	**24.4**	**21.3**
54	f	**30.2**	**28.5**	**27.6**	**26.6**	**24**	**21.1**
55	f	**30.3**	**29.9**	**26.5**	**19.6**	**21.1**	**18.8**
56	f	**30.2**	**29.9**	**28**	**26.5**	**19.3**	**20.8**
57	f	**30.3**	**32.1**	**31.1**	**31.3**	**21.4**	**20.8**
58	f	**35.2**	**34.4**	**29.4**	**27.6**	**24.3**	**21.9**
59	f	**29.8**	**25.5**	**23.7**	**20.2**	**21.7**	**16.2**
60	f	**30.8**	**29.5**	**19.9**	**19.4**	**20.8**	**19.5**
